# The Role of C-Reactive Protein and Neutrophil to Lymphocyte Ratio in Predicting the Severity of Odontogenic Infections in Adult Patients

**DOI:** 10.3390/medicina59010020

**Published:** 2022-12-22

**Authors:** Ovidiu Rosca, Bogdan Andrei Bumbu, Oana Ancusa, Serban Talpos, Horatiu Urechescu, Sorin Ursoniu, Vlad Bloanca, Marius Pricop

**Affiliations:** 1 Department XIII, Discipline of Infectious Diseases, “Victor Babes” University of Medicine and Pharmacy Timisoara, Eftimie Murgu Square 2, 300041 Timisoara, Romania; 2 Department of Dental Medicine, Faculty of Medicine and Pharmacy, University of Oradea, University Street 1, 410087 Oradea, Romania; 3 Department V, Discipline of Medical Semiology I, Faculty of General Medicine, “Victor Babes” University of Medicine and Pharmacy Timisoara, Eftimie Murgu Square 2, 300041 Timisoara, Romania; 4 Discipline of Oral and Maxillo-Facial Surgery, Faculty of Dental Medicine, “Victor Babes” University of Medicine and Pharmacy Timisoara, Eftimie Murgu Square 2, 300041 Timisoara, Romania; 5 Department of Functional Sciences, Center for Translational Research and Systems Medicine, “Victor Babes” University of Medicine and Pharmacy Timisoara, Eftimie Murgu Square 2, 300041 Timisoara, Romania; 6 Department of Plastic Surgery, “Victor Babes” University of Medicine and Pharmacy Timisoara, Eftimie Murgu Square 2, 300041 Timisoara, Romania

**Keywords:** inflammatory markers, disease severity score, odontogenic infections

## Abstract

*Background and Objectives*: Odontogenic infections (OI) represent a frequent cause of dental and maxillo-facial interventions, mostly due to late presentations or misdiagnosed complications. It is believed that the intensity of the immunoinflammatory response in OI is the main prognostic factor. Therefore, in this research, it was pursued to determine if the combination of C-reactive protein (CRP) and Neutrophil to Lymphocyte Ratio (NLR) (CRP-NLR) may serve as potential severity predictors in patients with odontogenic infections. *Materials and Methods*: A retrospective analysis on 108 patients hospitalized for odontogenic infections was conducted at the Department of Maxillofacial Surgery. Depending on the symptom severity scale, patients hospitalized with OI were divided into two equal groups based on infection severity (SS). *Results*: Patients with severe OI from Group B were associated more frequently with diabetes mellitus and smoking more often than those with a lower severity from Group A. In Group A, abscesses of odontogenic origin accounted for 70.4% of hospitalizations, while in Group B, abscesses and cellulitis were associated in 55.6% of cases (*p*-value < 0.001). The disease outcomes were more severe in Group B patients, where 22.2% of them developed sepsis, compared to 7.4% of Group A patients (*p*-value = 0.030). However, there was no significant difference in mortality rates. The SS and systemic immune inflammation index (SII) scores of Group B patients were substantially higher than Group A patients (13.6 vs. 6.1 for the SS score, *p*-value < 0.001), respectively, 2312.4 vs. 696.3 for the SII score (*p*-value < 0.001). All biomarker scores, including the CRP-NLR relationship, were considerably higher in Group B patients, with a median score of 341.4 vs. 79.0 in Group B (*p*-value < 0.001). The CRP-NLR association determined a 7.28-fold increased risk of severe OI. The receiver operating curve (ROC) analysis of CRP-NLR yielded an area under curve (AUC) value of 0.889, with high sensitivity (79.6%) and high specificity (85.1%), for predicting a severe odontogenic infection using biomarkers measured at hospital admission (*p*-value < 0.001). *Conclusions*: Therefore, it can be concluded that CRP-NLR is a reliable and affordable biomarker for determining the severity of odontogenic infections that may be included in other prognostic models for dental infections.

## 1. Introduction

Overall, the incidence of severe odontogenic infections is believed to be declining for a variety of reasons, including the availability of antimicrobials, innovations in healthcare delivery, and overall improvement in oral hygiene, leading to a decrease in mortality [1,2]. Infections of dental origin are rather prevalent, with some studies claiming they account for a significant percentage of antibiotic prescriptions. However, if left untreated, they might extend to the maxillofacial and cervical regions, hence posing a plethora of potential concerning issues [3,4].

It is crucial while treating patients with odontogenic infections to identify those that pose a high likelihood of developing serious consequences. These results may influence judgments on the dosage and effectiveness of therapy for some complicated cases. The strength of the immunoinflammatory response is believed to be a main prognostic factor [5]. Using factors derived from basic blood tests, several scores have been developed to predict the duration and severity of infections [6]. Such characteristics would be especially beneficial due to their quick availability and inexpensive cost. White blood cell (WBC) count, Neutrophil to Lymphocyte Ratio (NLR), and C-reactive protein are a few examples of (CRP) that were examined as objective assessment factors, but the findings were inconsistent [7,8,9].

Consequently, the white blood cell count (WBC) is a well-researched predictor of inflammation [10] with a half-life of 5–6 days. However, due to CRP’s fast peaks and falls, it is a more sensitive marker for the course of infection than WBC [11]. In addition, WBC count levels alone were inadequate to rule in or rule out the existence of infections [12]. However, increased levels are vague and have little diagnostic accuracy. For instance, WBCs have a minimal role in the diagnosis and severity assessment of head and neck infections [13]; their significance lies mainly in the evaluation of the patient’s response to therapy. In comparison to odontogenic infections, CRP is a better infection measure than WBC because its level rises more rapidly [14,15,16]. CRP is present in minute quantities in healthy persons, increases quickly with infection within a few hours [17], and then rapidly decreases when the inflammation subsides. Due to the tight relationship between the intensity and duration of acute infections, CRP is a sensitive indicator of inflammatory processes. Due to this, CRP is often utilized as a marker for odontogenic infection, which corresponds with hospital length of stay [18], while some authors believe it will never become a diagnostic tool on its own but can only be evaluated in conjunction with other clinical and pathological findings [19].

Instead, the NLR score, computed as the ratio between the neutrophil and lymphocyte counts detected in peripheral blood, is a valid indicator for detecting inflammatory status, bacteremia, and sepsis [20,21]. Because the early hyperdynamic phase of infection is characterized by a proinflammatory state mediated by neutrophils, an isolated increase in neutrophil count, and thus, an elevated NLR, can be observed in a variety of conditions, including bacterial or fungal infection, acute stroke, myocardial infarction, atherosclerosis, severe trauma, malignancies, post-surgical complications, or any condition that can activate systemic inflammatory response syndrome (SIRS) [22,23,24,25,26,27,28]. NLR is a simple, fast-reacting, and generally accessible indicator of stress and inflammation with high sensitivity but limited specificity [29]. It is frequently employed in practically all medical fields nowadays, including emergency care, surgical fields, and infections in the craniofacial area; however, it is the subject of relatively insufficient research [30].

Neutrophil to lymphocyte ratio (NLR) and C-reactive protein (CRP) levels are typically elevated in patients that develop abnormal inflammatory responses. To our knowledge, no studies have investigated so far the relationship between NLR and CRP with the severity of odontogenic infections. Therefore, it is believed that combining these two inflammatory scores would result in a more accurate disease-severity score, while the null hypothesis states that NLR-CRP association is an insignificant predictor of OI severity. In our investigation, we predicted that by combining the fast-rising characteristics of CRP with the high sensitivity of NLR for inflammation, it would be possible to obtain a measure with the capacity to predict the severity of odontogenic infections with great accuracy.

## 2. Materials and Methods

### 2.1. Study Design and Ethics

The study was designed as a retrospective cohort of patients admitted for odontogenic infections to the Maxillofacial Surgery Department of City Emergency Hospital Timisoara (SCMUT), affiliated with the Victor Babes University of Medicine and Pharmacy from Timisoara between January 2017 to April 2022. These data were collected from digital and paper records only with the patient’s agreement and the ethical approval obtained from the Ethics Committee of SCMUT with the approval number I-27098 from 14 October 2022.

### 2.2. Patient Selection Process

Patients above the age of eighteen were enrolled in the research. Infections of odontogenic origin were examined for inclusion according to the international classification of diseases (ICD-10) disease classification [31]. Patients whose medical records were incomplete were excluded from the research. Patients under the age of 18, pregnant women, and those with malignancy, immunodeficiency, or infections of origin other than odontogenic were excluded from the research, to avoid potential outliers in the levels of serum inflammatory markers. According to the Symptom Severity score (SS) presented in Table 1, eligible cases were divided into two groups based on the severity of the infection. The low-severity infection group consisted of mild to moderate infections, whereas the high-severity infection group included moderate to severe infections. At admission, the SS score of odontogenic infection created by Sainuddin et al. [30] and used in this study was calculated. Sepsis was defined by the recent guidelines in accordance with the sequential sepsis-related organ failure assessment score (SOFA) [32].

A convenience sampling method was employed to calculate the appropriate sample size. Considering the incidence of OI in the general population ranges between 0.05% and 0.1% [33,34], the computed ideal sample size was 34 patients, using a 99% confidence level and a 1 margin of error. Between January 2017 and April 2022, a total of 141 eligible patients identified with odontogenic infections were hospitalized at the Maxillofacial Surgery Department of the SCMUT. After deleting missing data and filtering by severity scores, 108 patients were eventually matched 1:1 by severity index and included in the study. The records were subsequently divided into two groups based on the primary anatomic space involved and the SS score: Group A consists of 54 individuals with a lower severity (SS score from 0 to 8 points); Group B consists of 54 patients with a greater severity (SS score from 9 to 16 points).

### 2.3. Data Collection and Variables

Demographic data and the patient’s medical history were collected. The hospital information system obtained the patients’ discharge reports, clinical evaluations, laboratory values, and imaging tests. Furthermore, routine blood samples, white blood cell count (WBC), hemogram indexes such as neutrophil and lymphocyte count, Neutrophil to Lymphocyte Ratio (NLR), and platelet count were evaluated. The variables considered for analysis comprised demographic data: age, gender, and place of origin, clinical presentation features (body temperature, trismus (mild, moderate, or severe), odontalgia (visual analog scale), mandibular pain (visual analog scale), dysfunctional disturbances of the masticatory system (mandibular dysfunction, headache, and unilateral chewing side)), edema, signs of obstruction (dyspnea, dysphagia), and signs of systemic infection (temperature >38.3 °C or <35.3 °C, heart rate > 90 bpm, respiratory rate > 20/min, blood pressure and WBC < 4 or >12 × 10³/μL) [35]. Routine blood sample on admission to the hospital: complete blood count, C-reactive protein, erythrocyte sedimentation rate (ESR), blood glucose levels, sodium and potassium, creatinine, and the glomerular filtration rate, blood urea nitrogen (BUN), aspartate transaminase (AST), alanine transaminase (ALT), clotting time, and swab culture with antibiogram. Research variables for serum parameters included the Neutrophil to Lymphocytes Ratio (NLR) obtained by dividing absolute Neutrophil and Lymphocyte counts.

### 2.4. Statistical Analysis

Data were obtained electronically and deidentified. Mean values and standard deviations (SD), *p*-values, and correlation coefficient “r” of the laboratory values were calculated using the statistical analysis software MedCalc (MedCalc Software bv, Ostend, Belgium). Variables were compared between group A and group B, including the laboratory tests mentioned above related to the Severity Score (SS) of odontogenic infections. The Mann−Whitney U test was applied to compare non-normally distributed means, while Student’s *t*-test was used to compare normally-distributed data. Chi-square and Fischer’s exact tests were applied to verify a possible difference between the two groups regarding variables described as proportionate values. Logistic regression analysis was applied to determine the association between CRP and NLR. The hazard ratio and adjusted odds ratios were determined for the assessment of CRP and NLR as predictors for infection severity (represented by SS score severity). The area under the curve (AUC) was plotted for CRP and NLR to determine their accuracy in predicting the severity of odontogenic infections. A *p*-value < 0.05 was considered statistically significant when comparing the study variables.

## 3. Results

### 3.1. Demographic Characteristics of the Study Population

**Figure 1:**
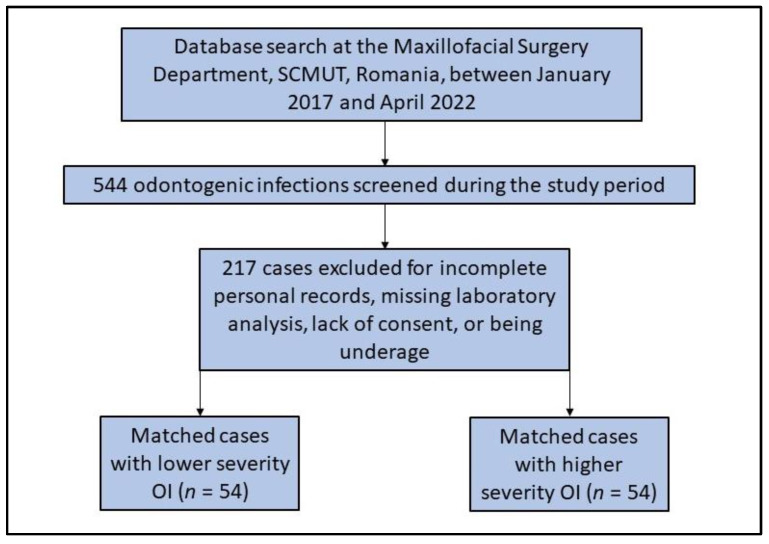
Patients’ inclusion flowchart.

In total, 544 patients diagnosed clinically and radiologically with odontogenic infections were admitted and hospitalized at the Maxillofacial Surgery Department, SCMUT, Romania, between January 2017 and April 2022. Only 108 patients met the inclusion criteria and were enrolled in the study, as described in Figure 1. The patients were further subcategorized according to the SS score into two groups as follows: Group A—the low-severity infection group with 54 patients whose severity score ranges from 0 to 8 points on the SS scale; Group B—the high-severity infection group including 54 patients with a severity score between 9 and over 16 points. Table 2 describes the comparison of background characteristics among patients with odontogenic infections. It was observed that men were more frequently involved with OI (55.6% in Group A and 66.7% in Group B). The mean age was 46.7 years in Group A (age range 18–81), compared with 51.7 years in Group B (age range 20–85), without a statistically significant difference (*p*-value = 0.150). However, the place of origin was significantly different between the study groups, with patients with more severe infections coming more frequently from rural regions (68.5% vs. 46.3%, *p*-value = 0.019). Additionally, patients with severe OI were more often affected by diabetes mellitus (*p*-value < 0.001), and smoking was more common in Group B compared to the group with lower severity infections (35.2% vs. 16.7%, *p*-value = 0.028).

### 3.2. Characteristics of Infection in the Study Population

Regarding the infection type in OI admitted to the hospital, 70.4% of them were abscesses in the lower infection cohort (Group A), while in Group B, 55.6% of infections were associations of abscesses and cellulitis (*p*-value < 0.001), as seen in Table 3. The most involved infection sites were the superficial lodges (40.7% vs. 48.1%), and peri-mandibular infections (25.9% vs. 33.3%), without statistically significant differences. Regarding disease outcomes, a total of 22.2% of patients in Group B developed sepsis, compared to 7.4% in Group A (*p*-value = 0.030), and four patients with severe OI were admitted to the ICU. However, mortality was not significantly different between the study groups (0.0% in Group A vs. 5.6% in Group B, *p*-value = 0.078). The median duration of hospitalization was significantly longer in patients from Group B, compared to Group A (12.0 days vs. 4.1 days, *p*-value < 0.001), in correlation with a higher frequency of severe complications in Group B (16.7% vs. 3.7%, *p*-value = 0.025).

The symptom severity evaluation presented in Table 4 identified a total of 32 (59.2%) patients with a SIRS score ranging from 0 to 1 in Group A. On the other side, Group B patients were only 13 (24.0%) within the 0–1 score range (*p*-value < 0.001). A severe trismus score was observed in 27 (50.0%) of patients from Group B, compared to only 9.3% in Group A (*p*-value < 0.001). Similar observations were noticed in the dysphagia score and fascial space score, where a statistically significantly higher prevalence of high severity was found in Group B patients. The prevalence of patients with odontogenic infections who were admitted with dehydration and significant comorbidities was significantly higher in Group B (29.6% vs. 5.6% in Group A, *p*-value = 0.001).

### 3.3. Risk Assessment in the Study Population

Table 5 presents the comparison of severity scores and biomarker scores among patients with odontogenic infections admitted to the hospital. It was observed that SS and SII scores were statistically significantly higher among patients in Group B (13.6 vs. 6.1, *p*-value < 0.001), respectively, 2312.4 in Group B compared to 696.3 in Group A (*p*-value < 0.001). All tested biomarker scores were significantly higher in Group B patients, including the CRP-NLR association, with a median score of 341.4, compared with 79.0 in Group B (*p*-value < 0.001).

**Figure 2:**
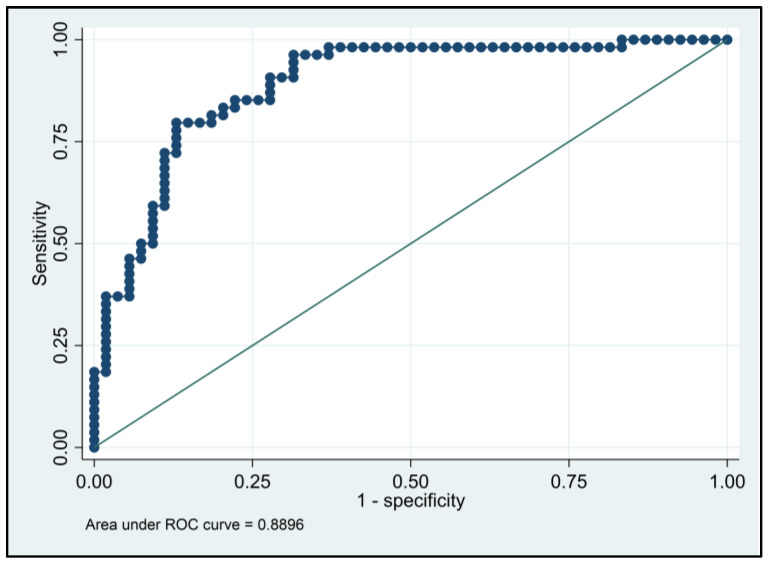
ROC curve analysis of CRP-NLR.

The logistic regression analysis presented in Table 6 describes the predictive of biological markers on the severity of odontogenic infections represented on the SS scale. It was observed that patients with an elevated WBC count had a 5.54 higher likelihood of severe OI, elevated neutrophils (OR = 7.10), elevated lymphocyte count (OR = 8.62), elevated NLR with an odds ratio of 4.46 (*p*-value < 0.001), high CRP levels with a 6.65 higher likelihood of severe OI, and lastly, the CRP-NLR association being responsible for a 7.28 higher risk (95% CI = 4.83–10.16). The ROC analysis of CRP-NLR resulted in a 0.889 AUC value (*p*-value < 0.001), with high sensitivity (79.6%) and high specificity (85.1%) for predicting a severe odontogenic infection using these biomarkers measured at hospital admission (Figure 2).

## 4. Discussion

### 4.1. Important Findings

The decision-making process in medicine incorporates clinical and laboratory considerations. Detecting an increase in acute phase reactants may assist the diagnostic interpretation of clinical symptoms in circumstances when an infection is suspected. In our investigation, CRP-NLR, which consists of CRP level at admission and NLR level at admission, was shown to have a more accurate ability to predict the severity of odontogenic infection. Initially, it was shown that a high CRP-NLR was substantially and strongly connected with high severity levels in odontogenic infection. Then, we examined the connection between CRP-NLR levels and WBC levels at admission in both severity groups and discovered that CRP-NLR had a greater predictive capability.

A better understanding of the inflammatory cascade has led to new discoveries and the identification of many mediators that, in combination with clinical symptoms, might serve as valuable infection indicators [36]. Bagul et al. [37] concluded in their study that CRP should be recommended as a monitoring marker for managing patients with fascial space infections of odontogenic origin, as it is a more sensitive indicator than WBC count and one of the best measuring tools for determining the infection control in these patients. In addition, John CR et al. [38] showed in their research analyzing indicators in patients with odontogenic fascial space infections that CRP should be suggested as a monitoring marker for the diagnosis of fascial space infection and for determining the response to treatment. In their research, Barreto et al. [39] found that the CRP test is a practical, easily accessible blood test that portrays the patient course and response to therapy more precisely than other commonly used indicators in oral and maxillofacial surgery.

Dynamic changes in NLR, on the other hand, predate the clinical condition by several hours and may alert doctors to an ongoing pathogenic process. Despite these benefits, NLR as a biomarker for assessing the progression of odontogenic infection has limited use. A recent meta-analysis [40] revealed that NLR was greater in non-survivors of sepsis than in survivors, and a larger NLR was linked with a worse prognosis in sepsis patients. Independent of the kind of operation (cardiac or abdominal), preoperative NLR levels are independent predictors of postoperative problems [41,42,43].

In addition, NLR may be used as a predictor for surgical treatment in submandibular abscesses [44] and as a recovery marker in odontogenic infection. Several investigations have demonstrated a correlation between NLR and the occurrence of pus, duration of hospital stay, and antibiotic dosage need [45]. In addition, the NLR value is constant and resistant to physiological and environmental factors, such as dehydration, physical exercise, and blood sample processing, that might influence test findings [46]. In their investigation, Dogruel et al. [47] determined that the NLR was related to hospitalization and antibiotic dosages in individuals with odontogenic infection. Incorporating the NLR into the CRP level has tremendous promise as a biomarker for odontogenic infection severity classification. Our objective was to determine whether CRP and NLR may serve as possible severity indicators in patients with odontogenic infections (OI).

According to several research, despite the increase in frequency, the patient features have remained basically unchanged [48]. The majority of patients were in their mid-30s, which is much younger than the majority of patients in our research, who were in their mid-40s. Furthermore, the amount of time between the beginning of symptoms and hospital presentation stayed comparable in both groups, and the percentage of patients who sought dental treatment prior to hospitalization remained surprisingly high at more than 40%, despite the fact that our study lacks this type of information. In addition, almost two-thirds of patients reported in previous studies had been orally administered antibiotics by their dentist or primary care physician before presenting to the hospital, a number that increased from 57% to 63%. Instead of seeking to cure the underlying cause, it is considered that an over dependence on antibiotics results in suboptimal patient care. Therefore, the need for prediction scores and algorithms is essential to determine the patients at risk.

### 4.2. Limitations of the Study

Our research has some important limitations and restrictions. To begin, the study was a single-center investigation of patients who had been admitted to the medical facility for odontogenic infections. Second, because of the retrospective design, we had to rely on the data from medical records; as a result, statistical analysis was susceptible to the risk of being inaccurate due to human error. Additionally, the retrospective study design impacts our results, as the research depends on the accuracy of both patient information tracking and the digital transcription of data from paper records. Limited by the retrospective design of our study, we could not perform a dynamic profile analysis of CRP and NLR, which may offer more helpful information. Other limitations are represented by country-specific features, since all patients were from Romania, and the oral hygiene can influence the severity of odontogenic infections. To provide more evidence in support of our results, further prospective research should be carried out.

## 5. Conclusions

This research aimed to determine whether there is a significant correlation between increased levels of inflammatory serum markers, as measured by the NLR and CRP, and the severity of odontogenic infections, as measured by the Symptom Severity score. The connection between these markers was discovered as an accurate predictor of OI severity. Thus, it can be concluded that CRP-NLR is a reliable and inexpensive biomarker to provide the severity of odontogenic infections that can be incorporated into other prognostic models to help determine the severity of odontogenic infections. Medical practitioners and their dental teams should be instructed to use the NLR-CRP score for the early identification and prognosis of severe odontogenic infections, hence potentially improving disease treatment choices.

## Figures and Tables

**Table 1:** The Symptom Severity score (SS) of odontogenic infections.

Criteria		Score	Max Score
Systemic Inflammatory Response Syndrome (SIRS)	Temperature > 38.3 °C	1	4
	Heart rate > 90 bpm	1	
	RR 20/min	1	
	WBC < 4 or >12 × 10^9^	1	
Trismus	Moderate < 2 cm	3	4
	Severe < 1 cm	4	
Dysphagia	Mild—able to swallow most foods	2	5
	Moderate—unable to swallow fluids	4	
	Severe—drooling saliva	5	
Collection in 1 fascial space	Low severity (canine, vestibular)	1	5
	Moderate severity (buccal)	2	
	High severity (all other spaces)	4	
Collection in 2 or more fascial spaces		5	
Sign of dehydration (↓BP/↑Urea/↓Skin turgor)		1	2
Comorbidities: diabetes mellitus, immunocompromised status, known or suspected chronic alcohol misuser		1	
Total Score			20

SIRS—Systemic Inflammatory Response Syndrome; BP—Blood Pressure; RR—Respiratory Rate; WBC—White Blood Cells.

**Table 2:** Comparison of background characteristics among patients with odontogenic infections.

Variables	Group A (*n* = 54)	Group B (*n* = 54)	Significance
Gender			0.236
Men	30 (55.6%)	36 (66.7%)	
Women	24 (44.4%)	18 (33.3%)	
Age, mean (mean ± SD)	46.7 ± 17.9	51.7 ± 18.1	0.150
Age range	18–81	20–85	NA
Place of origin			0.019
Rural	25 (46.3%)	37 (68.5%)	
Urban	29 (53.7%)	17 (31.5%)	
Smoking			0.028
Yes	9 (16.7%)	19 (35.2%)	
No	45 (83.3%)	35 (64.8%)	
Comorbidities			
Diabetes mellitus	10 (18.5%)	28 (51.9%)	<0.001
Obesity	31 (57.4%)	37 (68.5%)	0.231
Chronic kidney disease	14 (25.9%)	17 (31.5%)	0.523
Malignancy	5 (9.3%)	7 (13.0%)	0.540
Others	2 (3.7%)	4 (7.4%)	0.401

Data reported as *n* (%) and calculated using the Chi-square test and Fisher’s exact test unless specified differently; median and IQR values compared with Mann–Whitney u-test; IQR—Interquartile range.

**Table 3:** Comparison of infection characteristics among patients with odontogenic infections.

Variables	Group A (*n* = 54)	Group B (*n* = 54)	Significance
Reason for hospitalization			<0.001
Abscess	38 (70.4%)	17 (31.5%)	
Cellulitis	5 (9.3%)	7 (13.0%)	
Association of abscess and cellulitis	11 (20.4%)	30 (55.6%)	
Infection site			
Peri-maxillary	13 (24.1%)	10 (18.5%)	0.480
Peri-mandibular	14 (25.9%)	18 (33.3%)	0.399
Superficial lodges	22 (40.7%)	26 (48.1%)	0.438
Deep lodges	1 (1.9%)	2 (3.7%)	0.558
Fascial	5 (9.3%)	3 (5.6%)	0.462
Outcomes			
Sepsis	4 (7.4%)	12 (22.2%)	0.030
ICU admission	0 (0.0%)	4 (7.4%)	0.041
Duration of hospitalization, median (IQR)	4.1 (2.8)	12.0 (5.7)	<0.001
Severe complications	2 (3.7%)	9 (16.7%)	0.025
Mortality	0 (0.0%)	3 (5.6%)	0.078

Data reported as *n* (%) and calculated using the Chi-square test and Fisher’s exact test unless specified differently; median and IQR values compared with Mann-Whitney u-test; IQR—Interquartile range; ICU—Intensive care unit; SIRS—Systemic Inflammatory Response Syndrome.

**Table 4:** SS score differences among patients with odontogenic infections.

Variables	Group A (*n* = 54)	Group B (*n* = 54)	Significance
SIRS score			<0.001
0	14 (25.9%)	5 (9.2%)	
1	18 (33.3%)	8 (14.8%)	
2	10 (18.5%)	8 (14.8%)	
3	6 (11.1%)	19 (35.2%)	
4	1 (1.8%)	19 (35.2%)	
Trismus score			<0.001
Normal	30 (55.6%)	12 (22.2%)	
Moderate	19 (35.2%)	15 (27.8%)	
Severe	5 (9.3%)	27 (50.0%)	
Dysphagia score			0.028
Normal	5 (9.3%)	18 (33.3%)	
Mild	21 (38.9%)	16 (29.6%)	
Moderate	17 (31.5%)	29 (53.7%)	
Severe	0 (0.0%)	2 (3.7%)	
Fascial space score			<0.001
Low risk	39 (0.0%)	10 (18.5%)	
Moderate risk	23 (42.6%)	27 (50.0%)	
Severe risk	0 (0.0%)	8 (14.8%)	
Dehydration/Comorbid			0.001
No dehydration and comorbid	28 (51.9%)	13 (24.1%)	
Dehydration or comorbid	26 (48.1%)	22 (40.7%)	
Dehydration and comorbid	3 (5.6%)	16 (29.6%)	

Data reported as *n* (%) and calculated using the Chi-square test and Fisher’s exact test unless specified differently; SS—Severity Score; SIRS—Systemic Inflammatory Response Syndrome.

**Table 5:** Comparison of severity scores and biomarker scores among patients with odontogenic infections.

Variables	Group A (*n* = 54)	Group B (*n* = 54)	Significance
Severity scores, (mean ± SD)			
SS	6.1 ± 1.8	13.6 ± 3.9	<0.001 *
SII	696.3 ± 35.2	2312.4 ± 66.0	<0.001 *
Biomarker scores (median, IQR)			
WBC, (median, IQR)	9.34 (7.92–11.50)	12.02 (10.3–17)	<0.001 **
WBC_Ne, (median, IQR)	6.26 (4.68–10.23)	8.05 (6.73–10.61)	0.012 **
WBC_Ly, (median, IQR)	2.04 (1.41–2.7)	2.56 (2.06–3)	0.037 **
NLR, (median, IQR)	3.01 (2.10–4.83)	3.31 (3–4.37)	0.239 **
CRP, (median, IQR)	22 (9–47)	99 (86–118)	<0.001 **
CRP-NLR, (median, IQR)	79.00 (22.14–191.4)	341.47 (256.97–526.30)	<0.001 **

SD—standard deviation; SS—Severity Score; SII—Systemic Immune-Inflammation Index; WBC—White Blood Cells; WBC_Ne—White Blood Cells Neutrophils; WBC_Ly—White Blood Cells Lymphocytes; NLR—Neutrophil to Lymphocyte Ratio; CRP—C-Reactive Protein; * Student *t*-test; ** Kruskal–Wallis test; IQR—Interquartile Range.

**Table 6:** Hazard ratios and adjusted odds ratio for SS score calculated at admission for predicting SIRS and sepsis after odontogenic infections.

Variables	Risk (95% CI)	Significance
SS (dependent variable)		
WBC	5.54 (3.18–7.90)	<0.001
WBC_Ne	7.10 (5.19–9.01)	<0.001
WBC_Ly	8.62 (7.44–9.81)	<0.001
NLR	4.46 (3.53–5.40)	<0.001
CRP	6.65 (5.61–7.70)	<0.001
CRP-NLR	7.28 (4.83–10.16)	<0.001

Data were adjusted for age, comorbidities, and gender; WBC—White Blood Cells; WBC_Ne—White Blood Cells Neutrophils; WBC_Ly—White Blood Cells Lymphocytes; NLR—Neutrophil to Lymphocyte Ratio; CRP—C-Reactive Protein; CI—Confidence Interval.

## Data Availability

Data available on request.
